# Geometric accuracy of the MR imaging techniques in the presence of motion

**DOI:** 10.1002/acm2.12274

**Published:** 2018-02-01

**Authors:** Tarraf Torfeh, Rabih Hammoud, Tarek El Kaissi, Maeve McGarry, Souha Aouadi, Hadi Fayad, Noora Al‐Hammadi

**Affiliations:** ^1^ Department of Radiation Oncology National Center for Cancer Care & Research (NCCCR) Hamad Medical Corporation Doha Qatar; ^2^ Occupational Health & Safety Hamad Medical Corporation Doha Qatar

**Keywords:** geometric accuracy, motion, MRI, phantom

## Abstract

Magnetic Resonance Imaging (MRI) is increasingly being used for improving tumor delineation and tumor tracking in the presence of respiratory motion. The purpose of this work is to design and build an MR compatible motion platform and to use it for evaluating the geometric accuracy of MR imaging techniques during respiratory motion. The motion platform presented in this work is composed of a mobile base made up of a flat plate and four wheels. The mobile base is attached from one end and through a rigid rod to a synchrony motion table by Accuray^®^ placed at the end of the MRI table and from the other end to an elastic rod. The geometric accuracy was measured by placing a control point‐based phantom on top of the mobile base.

In‐house software module was used to automatically assess the geometric distortion. The blurring artifact was also assessed by measuring the Full Width Half Maximum (FWHM) of each control point. Our results were assessed for 50, 100, and 150 mm radial distances, with a mean geometric distortion during the superior–inferior motion of 0.27, 0.41, and 0.55 mm, respectively. Adding the anterior–posterior motion, the mean geometric distortions increased to 0.4, 0.6, and 0.8 mm. Blurring was observed during motion causing an increase in the FWHM of ≈30%. The platform presented in this work provides a valuable tool for the assessment of the geometric accuracy and blurring artifact for MR during motion. Although the main objective was to test the spatial accuracy of an MR system during motion, the modular aspect of the presented platform enables the use of any commercially available phantom for a full quality control of the MR system during motion.

## 1 INTRODUCTION

In the context of radiation oncology, respiration‐induced organ motion represents one of the main challenges for delivering the prescribed treatment plan. In the regions affected by breathing motion such as the thorax and the abdomen regions, the design of an appropriate motion management strategy ensures irradiating target tissues while sparing surrounding normal tissues.

Due to its excellent soft tissue contrast and nonionizing radiation employment, MRI is increasingly being used as an alternative modality to the four‐dimensional computed tomography (4D‐CT),^1^, ^2^ especially when determining patient‐specific breathing pattern for organs’ motion tracking during radiotherapy delivery. Furthermore, considerable effort is being invested in incorporating real‐time MRI during therapy delivery to develop new therapy techniques such as MRI‐guided high‐intensity focused ultrasound (HIFU) and MRI‐integrated Linear Accelerators (LINAC).^3^, ^4^, ^5^

As such, several MR motion management strategies have been clinically implemented including motion encompassing, motion controlling, or motion tracking where external or internal surrogates are used to obtain internal organs positions.^6^, ^7^, ^8^, ^9^, ^10^, ^11^, ^12^, ^13^, ^14^ These approaches use mostly 2D cine images acquired sequentially during some respiratory cycles and sorted retrospectively based on motion surrogates. The geometric accuracy of the MR images used for designing these motion management strategies is essential for ensuring the effectiveness of the radiation treatment. This geometric accuracy is altered mainly by the prolonged time required to collect sufficient data to form an image and can be observed in the form of blurring, ghosting, and signal loss in the image.^15^ Even though technological improvements have enabled faster imaging, this problem is far from being resolved due to biological and mechanical constraints.

Several studies have been carried out on assessing MR system related geometric accuracy in static mode.^16^, ^17^, ^18^, ^19^, ^20^, ^21^, ^22^, ^23^, ^24^, ^25^, ^26^ However, even though commercial platforms for motion management have been recently developed,^27^, ^28^ none of these studies dealt with the geometric accuracy of the MR sequences used for retrospective reconstruction of data acquired during breathing motion.

In this study, we present the design of a prototype of a modular platform allowing MR image acquisition during motion. In addition, tests conducted in order to evaluate 3D geometric accuracy as well as blurring artifact of balanced steady‐state gradient echo Cine MR Pulse sequence used with respiratory motion are described. This evaluation is performed using a point‐based phantom covering a 300 × 200 × 400 mm^3^ Field Of View (FOV).^18^ The geometric accuracy and the blurring artifact were evaluated for sequences acquired in the axial, sagittal, and coronal planes. MR images acquired with the phantom in static mode as well as during motion (dynamic mode) were evaluated. Finally, since the motion artifact is directly related to the time required to collect data and since the amount of time required to sample the signal differs between the frequency and phase‐encoding gradients, the impact of the direction of the encoding gradients was assessed by measuring distortion and blurring artifact before and after swapping phase and frequency‐encoding gradients.

## 2 MATERIALS AND METHODS

### 2.A The platform

**Figure 1:**
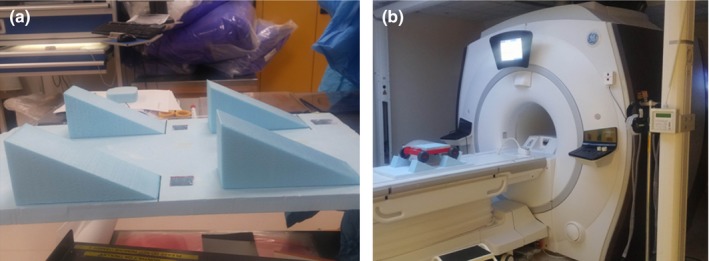
(a) The four ramps (b) the track placed on the ramps inside the MR scanner.

The motion platform presented in this work (Fig. 1) is composed of a mobile base made up of a flat plate and four wheels. The mobile base is attached from one end and through a rigid rod to a synchrony motion table by Accuray^®^ (Accuray Inc. Sunnyvale, CA, USA) placed at the end of the MRI table and from the other end to an elastic rod. When the synchrony motion table moves away from the phantom, the rigid rod is used to pull the base while elongating the elastic rod. On the other hand, when the motion table moves toward the phantom, the elongated elastic rod whose other end is attached to a fixed base pull back the mobile base toward its initial position.

This design allowed generating motion in the superior–inferior direction with a peak to peak movement of 25 mm and a cycle period of 5 s. In addition, four ramps of 28° made of foam were placed in front of each wheel. These ramps allowed the phantom to move maximum of 22.1 mm in the superior–inferior and 11.7 mm in the anterior–posterior directions simultaneously while maintaining its horizontal stability.

**Figure 2:**
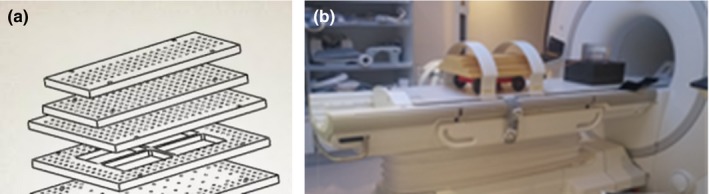
(a) View of the phantom showing the layers and (b) the phantom placed on a track attached to the motion motor positioned inside the MR.

The geometric accuracy was measured by placing on the top of the mobile base a phantom covering a Field of View of 300 × 200 × 400 mm^3^ and consisting of six layers of lightweight polyurethane foam material embedded with a matrix of Vitamin D capsules (Fig. 2). This phantom is a part of a larger phantom built by GE Healthcare and described in our previous work.^18^

### 2.B Clinical MRI geometric accuracy

MR scans were performed on a GE 1.5T MR‐SIM unit commissioned for radiation therapy (RT) planning, using GEM anterior array coil. Axial, sagittal, and coronal Cine MR slices of 8 mm thickness and a field of view of 370 mm were acquired. These scans consisted of 2D fast imaging employing steady‐state acquisition sequences with a flip angle of 50°, TE 1.5 ms, TR = 4.3 ms, a matrix of 512 × 512 pixel^2^, and a pixel bandwidth of 390.625 Hz/mm.

The studied sequences, summarized in Table 1, are identical to those used for RT applications.

**Table 1:** MR sequences used for the evaluation of the geometric distortion and blurring artifact

	Series	Acquisition details	Time
CINE	Axial FIESTA	TR = 4.5 ms. TE = 1.5 ms. Flip angle = 60^°^.195 Hz/Px. Thickness = 8 mm	4 min 7 s
	Coronal FIESTA		2 min 6 s
	Sagittal FIESTA		5 min 2 s

In addition to the geometric distortion, the blurring artifact was assessed by measuring the FWHM of the control points.

All the measurements were conducted on the phantom in static mode as well as in dynamic mode using the default phase and frequency‐encoding directions which are anterior–posterior for axial and sagittal acquisitions and left–right for coronal acquisitions. Furthermore, images were acquired after swapping the phase‐ and frequency‐encoding gradients, and all the measurements were then compared to those obtained with the default configuration. This procedure allowed assessing the impact of the direction of the encoding gradient on the geometric distortion and the blurring artifact for the studied sequences.

### 2.C Geometric accuracy calculation

**Figure 3:**
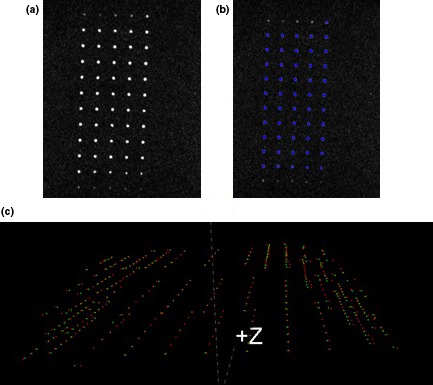
The process for the calculation of the geometric distortion. (a) MR image, (b) Automatic seed generation based on a threshold, and (c) 3D comparison of the MR (red) and CT (green) control points.

The geometric distortion was assessed for radial distances of 50, 100, and 150 mm from the center of the phantom. An in‐house Java‐based software module described in our previous work^18^ was used for automating the assessment of the geometric distortion. This module calculates the coordinates of each control point by measuring the center of mass (COM), then the algorithm corrects for gross phantom misalignment errors (translational and rotational errors), and finally establishes a one‐to‐one correspondence between the corrected control points in the MRI datasets and known positions obtained from the CT gold standard control points (Fig. 3).

**Figure 4:**
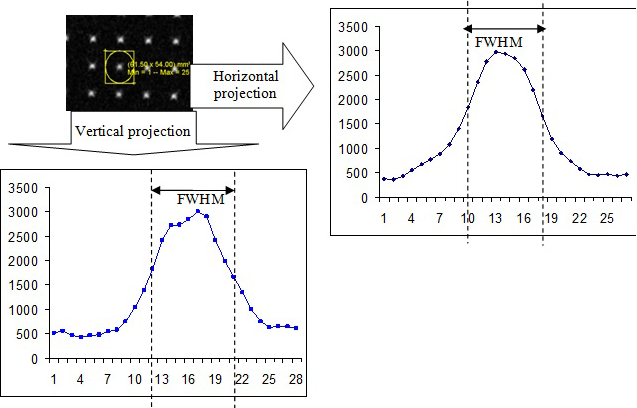
FWHM measurement. Original image of the ROI and the corresponding horizontal and vertical projections.

For the calculation of the FWHM, pixels within a square region surrounding each control point were projected in the horizontal and the vertical axes. The FWHM was calculated as the average FWHM measured in the two corresponding projections (Fig. 4).

### 2.D. Reproducibility

The reproducibility of our measurements has been investigated on four acquisitions, each with independent phantom setup. The geometric distortion and the blurring artifact were measured and compared on five axial, coronal, and sagittal CINE MR acquisitions.

## 3 RESULTS

### 3.A Geometric distortion

Table 2 shows the geometric distortion during both superior–inferior and anterior–posterior motion for the axial, coronal, and sagittal acquisitions. Up to a radial distance of 150 mm, the geometric distortion ranged between 0.2 and 1.4 mm for the axial acquisition, between 0.14 and 1.6 mm for the sagittal acquisition, and between 0.05 and 1.13 mm for the coronal acquisition.

**Table 2:** Distortion results for (a) axial, (b) sagittal, and (c) coronal acquisitions during both superior–inferior and anterior–posterior movements

Cine sequence			
Distortion (mm)			Radial distance
Mean	SD	Range	
(a) Axial			
0.65	0.5	0.0–0.9	50 mm
0.68	0.7	0.0–1.1	100 mm
0.95	0.6	0.0–1.4	150 mm
(b) Sagittal			
0.42	0.9	0.0–0.92	50 mm
0.78	0.9	0.0–1.27	100 mm
0.91	1.2	0.0–1.6	150 mm
(c) Coronal			
0.4	0.9	0.0–0.46	50 mm
0.54	1.32	0.35–0.82	100 mm
0.84	1.42	0.42–1.13	150 mm

**Figure 5:**
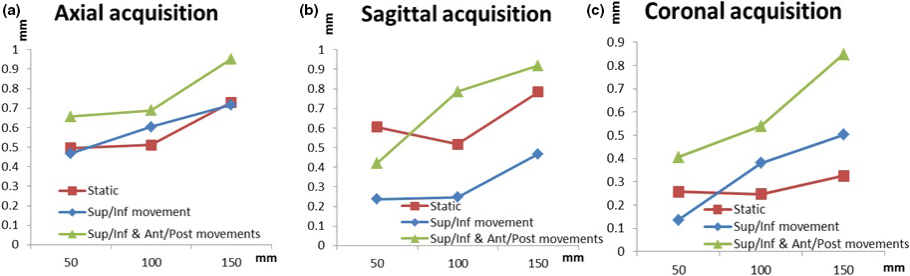
Geometric distortion measured with the phantom in static mode and during motion for (a) axial, (b) sagittal, and (c) coronal acquisitions, respectively.

Figure 5 shows the geometric distortion measured during superior–inferior motion alone and during both superior–inferior and anterior–posterior motions compared to the geometric distortion measured on the phantom in static mode for the axial, coronal, and sagittal acquisitions.

In the static mode and for a radial distance of 150 mm, the geometric distortion ranged between 0.2 and 0.9 mm for the axial acquisition, between 0.05 and 0.6 mm for the coronal acquisition, and between 0.14 and 0.87 mm for the sagittal acquisition. In the presence of superior–inferior movement alone and for a radial distance of 150 mm, the geometric distortion ranged between 0.1 and 1.2 mm for the axial acquisition, between 0.1 and 0.3 mm for the coronal acquisition, and between 0.1 and 0.9 mm for the sagittal acquisition. In the presence of both superior–inferior and anterior–posterior movements and for a radial distance of 150 mm, the geometric distortion ranged between 0.4 and 1.6 mm for the axial acquisition, between 0.3 and 1.13 mm for the coronal acquisition, and between 0.2 and 1.5 mm for the sagittal acquisition.

**Figure 6:**
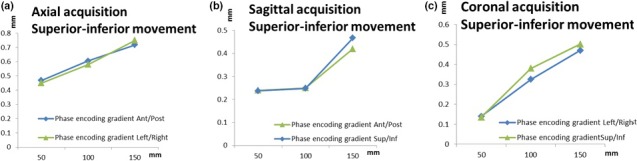
Geometric distortion before and after swapping the frequency‐encoding gradient during superior–inferior motion for the (a) axial, (b) sagittal, and (c) coronal acquisitions.

Figure 6 shows the geometric distortion before and after swapping the phase‐ and frequency‐encoding gradients with an only superior–inferior motion. The maximum variation in the mean geometric distortion was equal to 0.03, 0.05, and 0.06 mm for the axial, sagittal, and coronal acquisitions, respectively.

**Figure 7:**
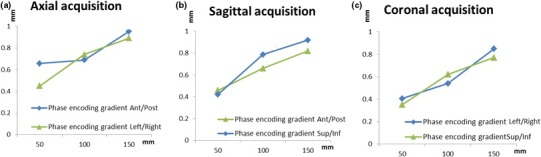
Geometric distortion before and after swapping the frequency‐encoding gradient during superior–inferior and anterior–posterior motions for (a) axial, (b) sagittal, and (c) coronal acquisitions.

Figure 7 shows the geometric distortion before and after swapping the phase‐ and frequency‐encoding gradients with a motion in both superior–inferior and anterior–posterior directions. The maximum variation in the mean geometric distortion was equal to 0.2, 0.8, and 0.7 mm for the axial, sagittal, and coronal acquisitions, respectively.

### 3.B Blurring artifact

Table 3 shows the mean FWHM measured during phantom motion and while the phantom is static for the axial, coronal, and sagittal acquisitions. In static mode and using the default phase‐encoding direction, the mean FWHM was equal to 6, 6, and 5 mm for the axial, coronal, and sagittal acquisitions, respectively. Including a superior–inferior movement, the mean FWHM increased to 6.5, 6.6, and 7.3 mm. Finally, in the presence of superior–inferior and anterior–posterior movements, the mean FWHM was equal to 6.6, 7.3, and 6.8 mm. After swapping the phase‐encoding direction, in the static mode, the mean FWHM was equal to 6.2, 5.6, and 5.1 mm. The same increase was noticed in the presence of superior–inferior movement obtaining a FWHM of 6.3, 7.6, and 8.6 mm. Finally, in the presence of both superior–inferior and anterior–posterior movements, the mean FWHM was equal to 6.2, 7.7, and 7.9 mm.

**Table 3:** The full width at half maximum for the axial, sagittal, and coronal acquisitions with and without motion, before and after the swapping of the phase‐encoding gradient

	FWHM (mm)			
	Phase‐encoding direction	Static	Superior–Inferior movement	Superior–inferior and anterior–posterior movements
Axial	Anterior–posterior	6	6.5	6.6
	Left–right	6.2	6.3	6.2
Sagittal	Anterior–posterior	6	6.6	7.3
	Superior–inferior	5.6	7.6	7.7
Coronal	Left–right	5	6.3	6.8
	Superior–inferior	5.1	8.6	7.9

### 3.C Reproducibility

The measurement of the geometric distortion was found reproducible while comparing the four acquisitions, with an average deviation of 0.13, 0.22, and 0.16 mm for the axial, coronal, and sagittal acquisitions, respectively. Furthermore, the average deviation of the blurring artifact was 0.34, 0.42, and 0.25 mm for the axial, coronal, and sagittal acquisitions, respectively.

## 4 DISCUSSION

There is an increasing interest in the utilization of magnetic resonance imaging in radiotherapy through planning, simulation as well as motion management. There are, however, some issues related to the use of MR in radiotherapy. The first relates to the lack of quantitative information about the attenuation of the tissues in MR as it is the case in CT imaging. A second major issue, which was studied in this work, is the potential error in its geometric accuracy, especially during motion. Several studies for assessing MR geometric distortion have been conducted; however, none of them treated the distortion in MR images during motion. In this study, we have presented a platform and software for assessing the MR geometric distortion during motion.

An MR compatible mobile base attached to a motion motor allowing a 22.1 mm motion in the superior–inferior direction and an 11.7 mm motion in the anterior–posterior direction has been presented and implemented. The modular aspect of this platform enables placing any commercially available phantom on top of the mobile base.

In our study, a control point‐based phantom was used and data has been collected allowing a detailed characterization of the geometric distortion during motion. The results obtained during this study showed that the geometric distortion measured on the phantom in static mode is comparable to the one measured during motion. An increase in the distortion measured during motion on the order of 0.04, 0.24, and 0.3 mm for radial distances of 50, 100, and 150 mm, respectively, was recorded. These results showed also that the direction of the frequency and phase‐encoding gradients has no significant impact on the geometric distortion. During the superior–inferior motion alone, the mean difference between the geometric distortions measured before and after swapping of the frequency‐ and phase‐encoding gradient was less than 0.6 mm while combining superior–inferior and anterior–posterior movements, these differences were less than 1 mm.

Regarding the blurring artifact, significant differences were observed between the phantom in static mode and during motion. Under superior–inferior motion alone, a FWHM increase in 13% was noticed. This increase reached 18% under both superior–inferior and anterior–posterior movements when compared to static mode.

For axial acquisition, an 8% difference was recorded between the FWHM measured on the phantom in static mode and the one measured during motion. This difference was 12% and 26% for sagittal and coronal acquisitions. This can be explained by the fact that small‐sized objects are more susceptible to blurring artifact than larger ones. Since ellipsoidal objects were used, the diameter within the coronal plane is smaller than those within axial and sagittal planes.

Finally, as expected, the blurring artifact was increased when the motion was in the same direction of the phase‐encoding gradient. This can be seen from the FWHM during the superior–inferior motion alone. For axial acquisition, the direction of phase‐encoding gradient will never be in the same direction as the movement (superior–inferior). This is translated by the low difference (3%) between the measured artifact before and after the swapping of the phase‐encoding gradient. On the other hand, for both sagittal and coronal acquisitions, an increase in 14% and 31% of the FWHM was recorded when the motion was in the same direction of the phase‐encoding gradient.

The platform presented in this study presents some design limitations, especially concerning the stability of the phantom during the motion. Misalignments of the phantom can occur during the motion due to the fact that the phantom is not attached to the mobile base. Furthermore, the potential interference of the materials with external sources of energy and its impact on the geometric distortion and blurring artifact should be evaluated. Future studies will be carried out by the authors in order to come up with a sufficiently stable and robust platform that can be commercialized.

These imperfections would have been important, in case the platform was used to design a motion strategy where the exact position of the platform should be known at any time. However, for the aim of this study, the motion reproducibility provided by the platform, the control point‐based phantom, and the misalignment correction performed by our software are sufficient for a detailed assessment of the geometric accuracy and blurring artifact for MR during motion compared to the measurements performed on the phantom in static mode. Furthermore, the use of widely available tools and materials such as a motion device and foam blocks as well as the detail of the platform design presented in this work make this prototype replicable by any institution. We believe that the work presented in this study, being the first to report a characterization of the MR geometric distortion during motion, represents an important first step toward a complete MR motion artifact investigation.

## 5 CONCLUSION

The platform presented in this work provide valuable tool for the assessment of the geometric accuracy and blurring artifact for both MR and CT modalities during motion. It allows generating motion within the superior–inferior and anterior–posterior directions with different cycle periods.

Our results showed no significant differences between the geometric distortion measured on the phantom in static mode and the one measured during motion. Regarding the phase‐encoding direction, even though no significant difference on the geometric distortion was found before and after the swapping of the phase‐encoding gradient, blurring artifact was noticed.

In addition to verifying the spatial accuracy of an MR system during motion, the modular aspect of this platform enables using any commercially available phantom instead of the control point‐based phantom which allows a full quality control of MR systems during motion.

## CONFLICT OF INTEREST

No conflicts of interest.
